# Contemporary effective population and metapopulation size (*N*_e_ and meta-*N*_e_): comparison among three salmonids inhabiting a fragmented system and differing in gene flow and its asymmetries

**DOI:** 10.1002/ece3.485

**Published:** 2013-02-01

**Authors:** Daniel Gomez-Uchida, Friso P Palstra, Thomas W Knight, Daniel E Ruzzante

**Affiliations:** 1 Department of Biology, Dalhousie UniversityHalifax, NS, Canada, B3H 4R2; 2 Parks Canada, Western Newfoundland and Labrador Field UnitPO Box 130, Rocky Harbour, NL, Canada, A0K4N0

**Keywords:** Effective population size, gene flow, metapopulation, *Salmo salar*, *Salvelinus fontinalis*, *Salvelinus alpinus*

## Abstract

We estimated local and metapopulation effective sizes (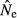 and meta-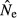) for three coexisting salmonid species (*Salmo salar, Salvelinus fontinalis, Salvelinus alpinus*) inhabiting a freshwater system comprising seven interconnected lakes. First, we hypothesized that 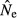 might be inversely related to within-species population divergence as reported in an earlier study (i.e., F_ST_: *S. salar> S. fontinalis> S. alpinus*). Using the approximate Bayesian computation method implemented in ONeSAMP, we found significant differences in 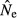 (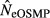) between species, consistent with a hierarchy of adult population sizes (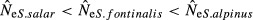). Using another method based on a measure of linkage disequilibrium (LDNE: 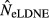), we found more finite 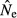 values for *S. salar* than for the other two salmonids, in line with the results above that indicate that *S. salar* exhibits the lowest 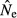 among the three species. Considering subpopulations as open to migration (i.e., removing putative immigrants) led to only marginal and non-significant changes in 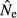, suggesting that migration may be at equilibrium between genetically similar sources. Second, we hypothesized that meta-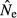 might be significantly smaller than the sum of local 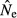
s (null model) if gene flow is asymmetric, varies among subpopulations, and is driven by common landscape features such as waterfalls. One ‘bottom-up’ or numerical approach that explicitly incorporates variable and asymmetric migration rates showed this very pattern, while a number of analytical models provided meta-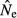 estimates that were not significantly different from the null model or from each other. Our study of three species inhabiting a shared environment highlights the importance and utility of differentiating species-specific and landscape effects, not only on dispersal but also in the demography of wild populations as assessed through local 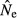
s and meta-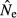
s and their relevance in ecology, evolution and conservation.

## Introduction

Multispecies comparisons of closely related taxa in landscape genetics studies have the potential to assist in validating models in theoretical population genetics and empirically assess their applicability, that is, the extent to which they capture the nuances of natural populations (Hänfling and Weetman 2006; Fraser et al. 2007; Beebee 2009). When model results are discordant, conclusions can be difficult to draw, but agreement, on the other hand, suggests that parameters inferred from the data may be robust to differential assumptions characterizing the theoretical models (e.g., Whitlock and Barton 1997). Studies making these types of comparisons may be especially informative for conservation and management of wild populations, as they can elucidate the general influence of life history aspects and demography on population genetics (Turner et al. 1996; Whiteley et al. 2004; Manier and Arnold 2006; Gomez-Uchida et al. 2009).

The effective size of a population (*N*_e_, Wright 1931) was defined to measure the rate of heterozygosity loss in a population, and is explicitly linked to migration rate (*m*) in the classical island model, through *F*_ST_ (Wright 1965). In addition to its importance at the interplay of population structure and dispersal, *N*_e_ is a central parameter in conservation biology as it is linked to the long-term viability and extinction risk of populations (Lande 1988; Frankham 1995). However, only recently have methods been developed that use genetic data to asse*s*s *N*_e_ independently of *m* (reviewed Wang 2005; Luikart et al. 2010). Such methods have been applied in numerous studies, mostly to reveal patterns of variable *N*_e_ within many taxa (fishes: Fraser et al. 2004; Gomez-Uchida et al. 2008; Palstra et al. 2009; reptiles: Manier and Arnold 2005; mammals: Kaeuffer et al. 2007; birds: Hoeck et al. 2010; see Palstra and Ruzzante 2008 for review). On the other hand, comparisons of *N*_e_ among different species occupying the same habitat have been sparse, even though these studies may provide evolutionary insights into previously documented ecological interactions (e.g., competition: Manier and Arnold 2005; predator-prey: Manier and Arnold 2006). Such analyses may also elucidate the role of life history in shaping variation in *N*_e_ in a shared common environment.

Most species naturally occur in networks of more or less interconnected populations (hereafter called meta-populations), thereby potentially biasing methods for estimating *N*_e_ that assume single closed populations. More importantly, this currently limits our understanding of the magnitude and behavior of *N*_e_ under typical conditions in the wild, both at the local scale of a population and at the global scale of a meta-population. Solutions have been proposed at different levels with various approaches (e.g., Fraser et al. 2007; Palstra and Ruzzante 2008; Waples and Do 2010). At the population level, approaches that simultaneously calculate contemporary *N*_e_ and *m* have gained popularity (Vitalis and Couvet 2001; Wang and Whitlock 2003; but see Waples and England 2011). Alternatively, some authors have attempted to identify immigrants and assess how they affect temporal estimates of contemporary *N*_e_ (Walter et al. 2009). Very few studies so far have explored the effects of gene flow on single-sample estimators of contemporary *N*_e_ (e.g., Palstra and Ruzzante 2011; Waples and England 2011). At the metapopulation level, the concept of effective metapopulation size (meta*-N*_e_), that is the effective size of a subdivided population, has been the focus of extensive theoretical work (e.g., Whitlock and Barton 1997; Nunney 1999). Wang and Caballero (1999) argued that meta*-N*_e_ can be smaller or larger than the sum of *N*_e_, depending on whether all subpopulations have equal variance in reproductive success. Recent empirical studies on salmonid fish have reported reductions in meta-*N*_e_ in the context of asymmetric gene flow and variable local *N*_e_ (Kuparinen et al. 2010; Palstra and Ruzzante 2011). These findings hence suggest that meta-*N*_e_ may indeed often be much smaller than would be predicted in relatively simple metapopulation models, but clearly more empirical estimates are required to validate this generalization.

**Figure 1:**
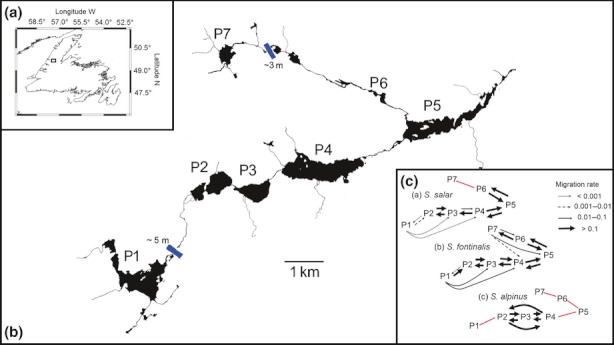
(a) Study site in the Upper Humber River, Gros Morne National Park (rectangle shows the area in western Newfoundland, Canada). (b) The population system: seven interconnected lakes locally referred to as “ponds” (P1–P7); P1 and P7 are found above waterfalls (in blue) of 5 m and 3 m, respectively, which are impassable for fish, whereas P2–P6 are found below waterfalls. River flows naturally from P1 to P5 and from P7 to P5. (c) Estimates of migration rate for (a) *Salmo salar*, (b) *Salvelinus fontinalis*, and (c) *Salvelinus alpinus* from a previous study (Gomez-Uchida et al. 2009) showed that these waterfalls represent barriers to dispersal and probably cause asymmetrical gene flow (arrows in log-scale), even though the magnitude was species-specific. Red lines indicate that no estimates of gene flow could be reliably obtained because samples were unavailable or insufficient (see text for details).

This study focuses on three salmonid fish species, Atlantic salmon (*Salmo salar*), brook trout (*Salvelinus fontinalis*), and Arctic charr (*Salvelinus alpinus*)*,* living in sympatry in a system of seven interconnected lakes in the Upper Humber River, Gros Morne National Park (Newfoundland, Canada: Fig. 1). All three species form discrete spawning aggregations within each lake, but with varying degrees of global population divergence (*F*_ST_: *S. salar* > *S. fontinalis* > *S. alpinus*; Table 1; Gomez-Uchida et al. 2009). Such levels of population divergence seem to be inversely related to population density as local mark-recapture studies indicate the abundance of *S. alpinus > S. fontinalis > S. salar* (Table 1; see also Ryan and Kerekes 1988; Anions 1994).

**Table 1:** Data set and population (genetic and ecological) parameters for a community of salmonids (*Salmo salar*, *Salvelinus fontinalis,* and *Salvelinus alpinus*) from the Upper Humber River, Gros Morne National Park (Newfoundland, Canada)

	*S. salar*	*S. fontinalis*	*S. alpinus*	Remarks
Data set				
Number of genotyped microsatellite loci	12	13	11	Details in Table S1–S4 from Gomez-Uchida et al. (2009)
Mean number of alleles per locus	13.2	8.6	18.2	Over all loci and populations
Number of subpopulations or ponds (names)	6 (P1–P6)	7 (P1–P7)	3 (P2–P4)	Minimum sample size of 20 to estimate *N*_e_
Number of genotyped individuals	456	706	301	Maximum of three loci with missing genotypes per individual*
Population				
Global population divergence – *F'*_*ST*_ (95% CI)	0.202 (0.200–0.205)	0.076 (0.058–0.089)	0.020 (0.012–0.025)	Standardized by heterozygosity
Global inbreeding coefficient – *F'*_IT_	0.202	0.088	0.012	Standardized by heterozygosity
Average immigration rate ( 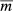 ± SE)	0.053 ± 0.025	0.061 ± 0.034	0.153 ± 0.050	Contemporary estimator using BIMr
Variance of dispersion distance (*σ*^2^)	0.026	0.023	–	No isolation by distance in *S. alpinus*
Adult population size	989	2021	12773	Mark-recapture estimate from Hardings pond (P2)

* Requirement for ONeSAMP (Tallmon et al. 2008).

Our goal in the present paper is twofold. We first test the hypothesis that contemporary effective population size will be inversely related to the degree of population structure with *S. alpinus* and *S. salar* exhibiting, respectively, the highest and lowest estimates of effective population size (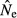) with *S. fontinalis* exhibiting an intermediate 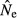. Secondly, we estimate meta*-N*_e_ (meta-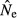) using a variety of models and then compare these estimates across models within species. Our second hypothesis, now related to meta-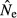, is that meta-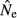 can be significantly smaller than the sum of local 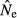
s if gene flow is asymmetric and varies among subpopulations (Tufto and Hindar 2003).

## Materials and Methods

### Study system and genetic data

The focal system consists of seven interconnected lakes or ‘ponds’ (P1–P7: Fig. 1) in the Upper Humber River, Gros Morne National Park, Newfoundland (details in Gomez-Uchida et al. 2009), where Atlantic salmon (*Salmo salar*), brook trout (*Salvelinus fontinalis*) and Arctic char (*Salvelinus alpinus*) naturally co-occur as non-anadromous landlocked populations, probably founded from anadromous populations that survived the Wisconsinan glacial maximum (10,000–12,000 BP) (Batterson and Catto 2001; Shaw et al. 2006).

The genetic data correspond to a subset of these ponds (hereby subpopulations) (Gomez-Uchida et al. 2009) including individual samples from *S. salar, S. fontinalis and S. alpinus* genotyped for 12, 13, and 11 microsatellite loci, respectively (Table 1). Most microsatellites assayed for *S. salar* and *S. fontinalis* were species-specific, but the majority of microsatellites assayed for *S. alpinus* originated from related salmonids (see Supporting Information in Gomez-Uchida et al. 2009). However, a high number of alleles per locus for *S. alpinus*, compared with the other two species (Table 1), suggest that ascertainment bias is probably negligible, if present at all. We set a minimum size of 20 individuals per population sample to estimate *N*_e_ and meta*-N*_e_, resulting in the exclusion of one subpopulation (P7) for *S. salar*, none for *S. fontinalis*, and two (P5 and P7) for *S. alpinus* and leaving 6, 7 and 3 (total 16) subpopulations available for analyses for each species, respectively (Table 1). Population genetics parameter estimates required for some models of meta-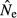, namely global estimates of divergence and inbreeding and contemporary estimates of migration, were taken from Gomez-Uchida et al. (2009).

### Effective population size (*N*_e_)

Firstly, contemporary 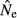 was estimated for each local subpopulation and species using ONeSAMP 1.2 (hereafter 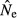
_OSMP;_ Tallmon et al. 2008). Briefly, the program employs approximate Bayesian computation to estimate the size of an ideal Wright-Fisher population (with no migration or selection) based on summary statistics calculated from the empirical data. The timescale to which the resulting *N*_e_ estimate applies is not explicit, but probably applies to recent generations (Luikart et al. 2010). We obtained four replicate estimates of 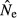 and their 95% credible limits (CL) under the following *prior* 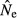 values: *N*_eMIN_ = 10 and *N*_eMAX_ = 2000. Although 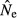
_OSMP_ appeared robust to different *N*_eMIN_ values across subpopulations and species (e.g., *N*_eMIN_ = 20, 40: authors' unpubl. results), changes in *N*_eMAX_ were not assessed, also because effective sizes in the three salmonids species were initially thought unlikely to be much larger than *N*_eMAX_ given the size of these small ponds.

Secondly, we estimated *N*_e_ plus parametric 95% confidence intervals (CI) using the linkage disequilibrium (LD) of LDNE (hereafter *N*_eLDNE;_ Waples and Do 2008). LDNE estimates reflect the number of parents that contributed to the sample (Waples 2005) and assume that LD at unlinked loci arises solely from genetic drift in an isolated population (Hill 1981; Wang 2005). Following Waples and Do (2010), we set the cut-off probability (*P*_crit_) for alleles of low frequency to be included in the estimation at *P*_crit_ = 1/2*S*, where *S* is the subpopulation sample size.

### Effects of immigration on 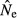
_LDNE_ and 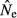
_OSMP_ assuming open subpopulations

The estimator methods implemented here assume that populations are closed to migration (Tallmon et al. 2008; Waples and Do 2008), but violation of this assumption may result in estimation bias (Vitalis and Couvet 2001; Palstra and Ruzzante 2011; Waples and England 2011). We thus conducted exploratory analyses to assess the impact of immigration on 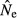, by identifying and excluding putative migrants from samples, and repeating the estimation of *N*_e_ in ONeSAMP and LDNE.

First-generation immigrants were identified in GENECLASS 2 (Piry et al. 2004) through Bayesian assignment of genotype likelihoods (Rannala and Mountain 1997). Here, individual genotypes were ranked according to the ratio *L*_h_/*L*_max_ that relates the likelihood of drawing genotypes from the populations in which they were sampled (numerator) with the maximum likelihood of such genotypes considering any of the study populations (denominator). We assumed that all sources of migrants were sampled (Paetkau et al. 2004) and attempted to minimize erroneous identification of migrants by setting the type I error at α = 0.01 and by graphically assessing likelihood scatter plots between recipient and source populations.

### Effective metapopulation size (meta*-N*_e_)

The estimation of meta*-N*_e_ from genetic data depends on the underlying model of spatial genetic structure (Wang and Caballero 1999). We have assumed that within our system, extinction and recolonization were infrequent and thus our model was more related to a “patchy” or “mainland-island” metapopulation than a classic Levins metapopulation (Koizumi et al. 2006). Here, we estimated meta*-N*_e_ assuming seven different models (Table 2), Firstly, we simply summed the various estimates of local *N*_e_ (Σ(*N*_e_)); this is our Null model. We then estimated the associated 95% confidence intervals following the standard equation SE(Σ (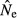) = sqrt [Σ (SE(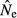)^2^)]. This was done taking into consideration the fact that lower and upper confidence intervals differed from each other. Then, we estimated meta*-*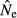 as defined by the Island (Wright 1931), Stepping Stone (Maruyama 1970), and Neighborhood models (Wright 1946; Maruyama 1971). These models assume that all subpopulations contribute equally to the next generation, but differ regarding assumptions on the spatial configuration and connectivity of subpopulations, yielding meta*-*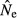 that should be larger than Null estimates (Table 2). Population parameters included in these models were taken from Table 1 or indirectly calculated from data obtained in Gomez-Uchida et al. (2009). Effective population density (*D*) for Neighborhood meta*-*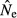 was obtained by dividing the sum of subpopulation 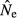
s by the linear habitat length (*L* = 11 km); the variance of dispersion distance (σ^2^) was estimated from the slope (*b*) of the isolation-by-distance relationship described in (Gomez-Uchida et al. 2009) according to 4*D*σ^2^ = 1/*b* (Rousset 1997). Stepping Stone and Neighborhood meta-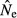 were ignored for *S. alpinus*, because we found no indications that an isolation-by-distance model was applicable to this species (Gomez-Uchida et al. 2009). Second, we calculated Interdemic meta-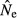
*;* this model predicts meta-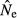 can be smaller than the Null estimate when accounting for variable fitness among subpopulations (Nunney 1999). Third, we explored Spatio-temporal meta-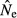
by Kobayashi and Yamamura (2007), which provides similar expectations to Island meta-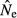, but is centered on the average number of contemporary immigrants (Table 2). Finally, we used the empirical ‘bottom-up’ approach of Tufto and Hindar (2003), which combines subpopulation 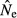
s (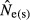
calculated previously using ONeSAMP) with unidirectional migration rates (estimated using BIMr) to calculate T&H meta-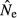. Here, we minimized the eigen-value of the resulting metapopulation matrix employing an R package (R Development Core Team 2010) developed by J. Tufto (available from http://www.math.ntnu.no/∼jarlet/migration/). Given sample size limitations (see above), meta-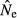 for *S. fontinalis* was estimated considering all seven subpopulations and for *S.salar,* it was estimated over six subpopulations (omitting P7). *Salvelinus alpinus* was collected in adequate numbers only in three ponds (P2–P4); therefore, meta-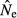 for this species was limited to those three ponds (see Results).

**Table 2:** Models of spatial genetic structure and estimation of effective metapopulation size (meta-*N*_e_) or the size of an idealized population with the same rate of inbreeding observed in the subdivided population under study

Name	Model*	Expectations	Reference
Island	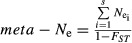	meta-*N*_e_ > 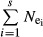 unless *F*_*ST*_ = 0; inequality increases with *F*_ST_ (divergence)	Wright (1943)
Stepping stone (circular)	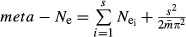	meta-*N*_e_ > 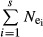 ; inequality increases with decreasing average immigration rate ( 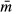 )	Maruyama (1970)
Neighborhood (linear)	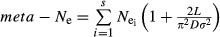	meta-*N*_e_ > 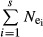 ; inequality increases with increasing length of the habitat (*L*), but decreases with increasing population density (*D*)	Wright (1946) Maruyama (1971)
Interdemic	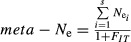	meta-*N*_e_ < 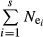 ; inequality increases with metapopulation inbreeding	Nunney (1999)
Spatiotemporal	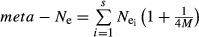	meta-*N*_e_ > 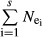 ; inequality increases with the average number of migrants per generation 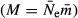	Kobayashi and Yamamura (2007)

* Metapopulation parameters: *s* = number of subpopulations/ponds; 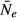 = average subpopulation *N*_e_; F_ST_ = global genetic divergence among subpopulations; 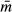
= average immigration rate; *D* = linear population density (individuals/km); *L* = length of habitat (km); *σ*^2^ = variance of dispersion distance; F_IT_ = coefficient of global (metapopulation) inbreeding.

### Statistical analyses and hypothesis testing

We assessed the null hypothesis of no correlation between 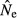 and sample size using Kendall's τ correlation coefficient and two-tailed exact probabilities, given the uncertainty as to whether the data were drawn from a bivariate normal distribution or not. Such correlations permitted to evaluate any dependence of 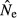 on sample size using ONeSAMP as LDNE provides unbiased estimates (Waples and Do 2008). To evaluate differences in *N*_e_ estimates among species, the null hypothesis of no differences was tested using nonparametric Kruskall–Wallis tests for multiple species comparisons and Mann–Whitney one-tailed tests for pairwise species comparisons. When replicates were available (e.g., ONeSAMP), we compared the harmonic means to minimize the contribution of overly distinct replicates. All tests were implemented using R 2.13.2 (R Development Core Team 2010).

## Results

### 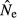: Comparisons between estimators and species


**Figure 2:**
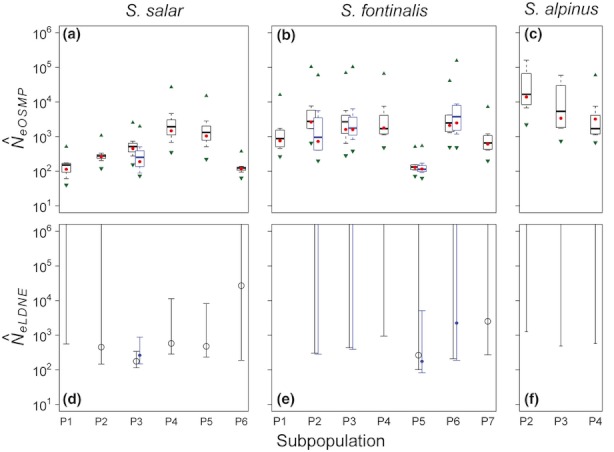
Estimates of effective population size (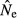) and their uncertainty for *Salmo salar* (left column), *Salvelinus fontinalis* (middle column), and *Salvelinus alpinus* (right column) using two estimators, 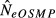 (top row; boxplots) and 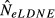 (second row; empty circles). Blue boxplots or filled circles are estimates of 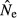 following exclusion of immigrant genotypes (see text for details), thus assuming populations open to gene flow. Red dots in boxplots are the harmonic means among four 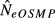 replicates, whereas green triangles represent the lowest (inverted triangles) and highest 95% CIs among four 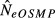 replicates. Missing or out-of-bound empty or filled symbols, upper 95% CIs, or both, suggest infinitely large values.

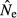
 was estimated for 16 species/subpopulation combinations (Fig. 1). While all 16 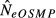 values were finite, their magnitude differed across replicates, in some cases considerably so (Fig. 2a–c). For 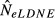, on the other hand, only seven or eight estimates were finite depending on whether immigrants were included or excluded, most of which were concentrated among *S. salar* subpopulations (Fig. 2d–f). The range of values (box plots) for 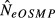 replicates was narrow for *S. salar*, intermediate for *S. fontinalis* and wide for *S. alpinus* (Fig. 2a–c). Two 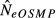 replicates exhibited extremely high upper confidence limits (in the millions of individuals). On the other hand, only four upper 95% CIs were finite with LDNE (Fig. 2). Finite 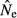 estimates were generally poorly correlated with sample size (*S. salar,* 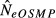 τ = 0.69, *P* = 0.06; *S. salar*, 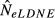 τ = −0.10, *P* = 0.80; *S. fontinalis*, 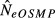 τ = −0.14, *P* = 0.77; no correlation test was performed with 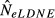 for *S. fontinalis* and no correlation tests were attempted for *S. alpinus* with only three estimates available). Comparisons of 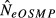 harmonic means among the three salmonids indicated significant differences (Kruskal–Wallis χ^2^ = 8.8, df = 2, *P* = 0.01); furthermore, one-tailed Mann–Whitney pairwise tests were consistent with a hierarchy of 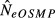 values as proposed (*S. salar* < *S. fontinalis*: *P* ≤ 0.05; *S. fontinalis* < *S. alpinus*: *P* ≤ 0.008; *S. salar < S. alpinus*: *P* ≤ 0.01). Complete details on sample sizes, 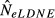, and 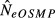 can be found in Table S1 (Supporting Information).

### Immigrant genotypes and their impact on 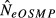 and 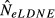 (open subpopulations)

Immigrant genotypes that could be identified ranged between 1 and 3 among subpopulations and across species (Table S1). In four out of five *S. fontinalis* subpopulations, the likely source of immigrants was either P1 or P7, both ponds found above waterfalls (Fig. 1), whereas all three *S. salar* individuals identified as immigrants in P3 probably originated from P5 (Table S1). The weak population structure observed in *S. alpinus* prevented the reliable identification of potential immigrants for this species.

Both estimators – 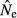
_OSMP_ and 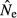
_LDNE_ – suggested that the changes in 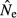 after exclusion of immigrants were negligible and non-significant (Fig. 2, Table S1).

### Meta- 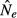


**Figure 3:**
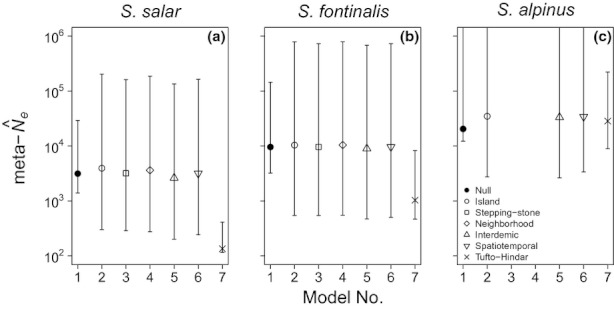
Estimates of *meta-* 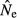
from the null model (sum of subpopulation *N*_e_'s) and six metapopulation models (island, stepping stone, neighborhood, interdemic, spatiotemporal, and Tufto-Hindar: see legend) for (a) *Salmo salar*, (b) *Salvelinus fontinalis*, and (c) *Salvelinus alpinus*. Stepping stone and neighborhood models require the assumption of isolation-by-distance and were therefore omitted for *S. alpinus* (see text for details).

Given the generally low precision of 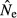
_LDNE_*,* the estimates of meta*-N*_e_ were based only on 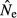
_OSMP._ Meta-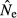 differed across species by up to three orders of magnitude; it was smallest in *S. salar,* intermediate in *S. fontinalis,* and largest in *S. alpinus*. Moreover, these qualitative differences across species were constant regardless of the spatial population model assumed (Fig. 3). Indeed, the differences in meta-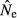 between estimator models were very small, with one notable exception: the meta-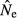
obtained with the Tufto and Hindar (2003) model, which were always much smaller than any of the other meta-*N*_e_ estimates. In fact, T&H meta-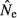 for *S. salar and S. fontinalis* were similar to the 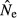
_OSMP_ estimates obtained for both species in one subpopulation, P1 (Table S1 and Table 3).

## Discussion

In this study, we estimated the local and metapopulation effective sizes of three salmonid fish species living in sympatry in a spatially fragmented system of seven interconnected subpopulations and exhibiting marked differences in demographic attributes as measured by the F_ST_ analysis of Gomez-Uchida et al. (2009). First, putative immigrants had negligible effects on estimates of local effective population size (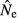) and the estimates generally fit our expectations based on demography and life history with the local effective size of *S. salar < S. fontinalis < S. alpinus*. Second, we implemented six approaches to estimate effective metapopulation size (meta*-*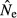) and compared these estimates with the sum of local 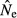 ∑(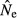), a model we defined as Null. The majority of meta-*N*_e_ estimators yielded results indistinguishable from the sum of local sizes, despite considerable variation in population structure among the species. This suggests that these analytical models may not fully capture the complexity of natural systems or simply are not well suited for application to empirical data. On the other hand, the estimates obtained with the Tufto and Hindar (2003) approach [T&H meta-Ne] differed substantially from the other models and were strongly influenced by connectivity patterns inferred from the genetic data. The linear property of our system, combined with asymmetric dispersal facilitated by the presence of waterfalls, may thus have resulted in strong reductions in meta-*N*_e_ in line with other recent empirical work and theoretical expectations. Below, we review some caveats regarding the methods used to estimate contemporary local 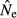 and discuss how immigration seemed to have negligible impacts on 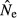; we then argue how possible differences in life history (migration and abundance) reflect on differences in average local 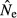
s among species. We subsequently consider estimates of meta-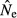 and the similarities and differences among models within species. We finish by explicitly discussing our results in the context of known species differences in life history (migration as measured by gene flow) and abundance.

### ONeSAMP and LDNE: Caveats and patterns across species

The two methods differed in performance within the scope of our study. Firstly, they differed in the number of finite 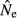 estimates they provided: all point estimates of effective population size were finite when estimated with ONeSAMP, but only seven or eight (depending on whether immigrants were included or excluded) were finite when estimated with LDNE. As the estimation of contemporary *N*_e_ depends largely on the genetic drift signal, which is stronger in smaller populations (Fraser et al. 2007; Palstra and Ruzzante 2008; Waples and Do 2010), it should not be surprising that both approaches provided finite values only for *S. salar*, the species with the lowest abundance and historical census size (Ryan and Kerekes 1988; Anions 1994).

Secondly, the two methods apply to different time scales: while LDNE uses linkage disequilibrium to estimate the effective size in the parental generation (Waples and Do 2008), ONeSAMP uses several metrics, including linkage disequilibrium (Tallmon et al. 2008) to estimate effective size over the last few, but undefined number of generations (Luikart et al. 2010; see also Skrbinsek et al. 2012). This is the likely cause of the discrepancy of results for *S. salar* in the headwater pond P1 (Fig. 2 and Table S1): the M-ratio test (Garza and Williamson (2001), which is considered in ONeSAMP, suggests that a bottleneck has occurred in this population (Gomez-Uchida et al. 2009), but this test statistic is not considered in the LDNE method. It should be noted, however, that the removal of first-generation migrants prior to the estimation of effective size does not necessarily completely eliminate the influence of migration if migration took place in previous generations. Such earlier migration could be affecting *N*_e_ particularly when estimated with ONeSAMP.

A third caveat regarding our replicated estimates of *N*_e_ is that they were all obtained with priors set at *N*_emin_ = 10 and *N*_emax_ = 2000. For *S. salar,* only few point estimates (3 out of 24) exceeded 2000 (Table S1), suggesting that the true *N*_e_*s* for this species falls mostly within this range. On the other hand, approximately one-third of the replicates for *S. fontinalis*, and two-thirds of the replicates for *S. alpinus* were higher than the N_max_ prior (See Table S1). These results suggest that for these two species, and particularly for *S. alpinus,* the true *N*_e_ values may indeed be larger than 2000 and hence the program had difficulty estimating them (David Tallmon, personal communication). Our caveats notwithstanding, Beebee (2009) also found that *N*_e__OSMP_ showed higher precision than *N*_e__LDNE_ for natterjack toad (*Bufo calamita*) populations for which *N* varied between 2 and 500, that is, within the range of the estimates found for *S. salar* in our study.

Additionally, age-structure and overlapping generations can significantly complicate the estimation of *N*_e_ (Palstra et al. 2009). We used samples consisting of different ages, which probably reflect a quantity intermediate between *N*_e_ and *N*_*b*_, the annual number of breeders (Waples 2006). The samples analyzed in this study may include three or four cohorts based on length ranges (*S. salar*: 78–248 mm; *S. fontinalis*: 50–230 mm; *S. alpinus*: 87–157 mm, authors' unpublished results) and length-at-age data from earlier surveys in Gros Morne National Park (Rombough et al. 1978; Barbour et al. 1979; McCairns 2004). The number of cohorts in our samples thus approximates the mean generation length (*G*) based on estimates of the mean age of reproductive parents in this system (*S. salar G* = 4.5: (Rombough et al. 1978); *S. fontinalis G* = 4.5: (McCairns 2004); *S. alpinus G* = 3.5: (Barbour et al. 1979). Under such sampling conditions, Waples and Do (2010) suggested that *N*_b_ ≍ *N*_e_, even though other researchers have opted to report their estimates as *N*_b_ (e.g., (Beebee 2009) or apply the correction *N*_e_ = *GN*_b_ (e.g., Ardren and Kapuscinski 2003; see also Robinson and Moyer 2012). Therefore, it may be recommendable to complement single- with two-sample temporal estimates, especially methods tailored to gauge allele frequency shifts between cohorts, which require individual ages (Palstra et al. 2009; Waples and Do 2010).

To summarize, the local effective sizes for the *S. alpinus* and *S. fontinalis* populations studied here are likely larger than can reliably be estimated given the sample sizes (S∼ 90–100) and numbers of microsatellite loci (L∼12–13) available, but this limitation appears to be stronger for LDNE than for ONeSAMP. Overall, *N*_eOSMP_ may therefore appear to be more general than *N*_eLDNE_, due to the rationale and flexibility of its methodology (prior specifications, simulation approach). On the other hand, the main drawback of ONeSAMP may be its undefined temporal reference (Luikart et al. 2010; see also Skrbinsek et al. 2012), thereby reducing its utility for genetic monitoring. Ultimate choice of *N*_e_ estimator should be guided by the specific aims and goals of each study.

### Impacts on 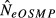 and 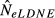 of accounting for gene flow

We attempted to account for the effect of gene flow among subpopulations by re-estimating effective population sizes after eliminating putative immigrants. This approach was implemented with the two species for which immigrants were identified: *S. salar* and *S. fontinalis*. Although in all cases the point estimates of *N*_e_ decreased after the removal of putative migrants, these decreases were minor and non-significant (Fig. 2), suggesting that migration may be sustained and at equilibrium between genetically similar sources (Waples and England 2011). We reiterate, however, that the removal of first-generation migrants does not necessarily completely remove the influence of migrants in the estimation of *N*_e_. As linkage disequilibrium between physically unlinked loci decreases by 50% each generation, any LD caused by migration in the grand-parental or great-grand-parental generation would probably still influence the estimation of *N*_e_ with either method.

### Comparisons of *N*_e_ among species

Despite the limitations imposed by the relatively large population sizes for two of our study species, both approaches provided similar insight into putative links between population abundance, life history and *N*_e_. Pairwise comparisons highlighted that *S. alpinus* probably has higher *N*_e_ than the other two species, matching expectations based on its life history and abundance (e.g., high population abundance and connectivity (Gomez-Uchida et al. 2009). Landlocked *S. alpinus* from Gros Morne National Park aggregate in large numbers in only three subpopulations of the watershed (Gomez-Uchida et al. 2009) and are rare or even absent in other lakes within the system (authors' personal observations). Arctic charr were described as having slow growth, low fecundity, and early age at maturity by previous authors (3 years, Rombough et al. 1978), all likely consequences of intense inter- and intraspecific competition in low-productive acidic lakes (Rombough et al. 1978; Anions 1994). The adult census estimate of *N ˜* 13,000 for *S. alpinus* within one pond (P2) based on mark-recapture (Anions 1994) suggests that *N*_e_ for this species should indeed be large, at least in the order of a few thousands, even though imprecision of this effective population size estimate may be an issue. Massive densities of so-called dwarfed or ‘stunted’ *S. alpinus* populations have also been described in Norwegian oligotrophic lakes (Jansen et al. 2002; Persson et al. 2007). Conversely, the estimated adult census size for *S. salar* within P2 was *N* ∼ 1000 (Anions 1994), a number that is consistent with the estimate of effective size obtained for this species within this pond (Fig. 2). We found significant differences in 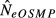 between *S. salar* and *S. fontinalis*, two salmonids that showed a similar range of distribution in the watershed (in all subpopulations but one, P7) and comparable average immigration rates, but a nearly twofold difference in adult population sizes, a finding that is consistent with our hypothesis.

### Effective metapopulation size (meta-*N*_e_)

Theory predicts that, under the simplified and restrictive assumptions of the island model, the effective size of a metapopulation will be higher than the sum of subpopulation *N*_e_ (reviewed in Wang and Caballero 1999). Unsurprisingly, these predictions are borne out in the analyses that are explicitly underpinned by those assumptions. The difference between (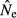) and island model meta-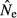 was proportional to the degree of fragmentation, with *S. salar* exhibiting the most pronounced spatial structure (highest *F*_ST_), and also the largest difference between (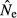) and meta-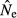. On the other hand, we find a remarkable consistency across estimates of meta-*N*_e_ for the majority of analytical models considered, despite the considerable range of population structuring observed (0.020 ≤ *F*_ST_ ≤ 0.202). Moreover, within species, these models provided highly similar estimates of meta-*N*_e_ that did not deviate strongly from ∑(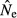). Although these models make very different assumptions about the metapopulation and consider different genetic parameters, in practice, their meta-*N*_e_ estimates can often be reasonably approximated by simply summing individual population *N*_e_ estimates. Further testing of these models in different systems and species is naturally required to assess the validity of this observation.

The notable exception to this pattern was the meta-*N*_e_ estimate obtained with the empirical method of Tufto and Hindar (2003). This approach provided estimates, which were considerably smaller than ∑(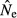) as expected under conditions of strongly asymmetric gene flow among metapopulation components (Whitlock and Barton 1997; Nunney 1999). Indeed, the Tufto and Hindar (2003) model makes fewer restrictive assumptions about metapopulation dynamics than the other methods, allowing for consideration of heterogeneity in the size of metapopulation components and gene flow among these components. For instance, in the present study, both *S. salar and S. fontinalis* were very abundant in one of the headwater ponds or subpopulations (P1), which lie above a waterfall that impedes upstream migration. This is also the case for P7, another headwater subpopulation where *S. fontinalis* was found in great numbers. Under such conditions, the reproductive contributions among demes may diverge strongly from expectations of equality. Indeed, for both species, meta-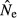 was very similar to the estimate of local population effective size in the upstream (headwater) population. Hence, genetic diversity in this (linear) system, for at least two study species, may be largely controlled by the diversity that exists in the ‘upstream’ source population. This inference is compatible with Pringle et al. (2011), who suggested that in systems with asymmetric gene flow, the upstream edge can influence genetic diversity throughout the entire species' range. Conversely, in *S. alpinus,* we documented populations with typically high abundance (Table S1) and relatively high and symmetric migration patterns among populations, and meta-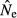 was intermediate between the smallest and largest subpopulations 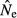. Therefore, our results provide further empirical support for the theoretical expectation (Whitlock and Barton 1997) that strong inequality among demes in contribution to the shared gene pool can reduce the effective size of a subdivided population.

To synthesize, in this study, we have estimated local or subpopulation effective sizes, as well as gene flow among them, in three sympatric species of salmonids fishes. We then compared the sum of these local effective sizes ∑(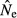) to the meta-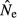 obtained under the Tufto and Hindar (2003) model. The relative magnitude of T&H meta-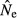 across species was consistent with estimates of relative abundance available for one of the ponds at a different point in time (Ryan and Kerekes 1988; Anions 1994). Moreover, this pattern mirrors the hierarchy and degree of asymmetry in population connectivity described in Gomez-Uchida et al. (2009). As our study species shared a common environment, differential riverscape effects are unlikely to have shaped these patterns. Rather, the apparent relation between connectivity patterns and census population sizes across species which share a common environment, provides further support for the notion that density-dependent dispersal may play an important role in the maintenance of genetic diversity in aquatic systems and in metapopulations in general.

## Supporting Information

**Table S1.** Brook trout (*Salvelinus fontinalis*), Atlantic salmon (*Salmo salar*), and Arctic charr (*Salvelinus alpinus*) estimates of effective population size per pond (Ne) plus 95% credible limits (CL) or confidence interval (CI) using ONeSAMP and LDNE, respectively.
